# Relationship Between Soil Type and N_2_O Reductase Genotype (*nosZ*) of Indigenous Soybean Bradyrhizobia: *nosZ*-minus Populations are Dominant in Andosols

**DOI:** 10.1264/jsme2.ME14130

**Published:** 2014-12-03

**Authors:** Yoko Shiina, Manabu Itakura, Hyunseok Choi, Yuichi Saeki, Masahito Hayatsu, Kiwamu Minamisawa

**Affiliations:** 1 Graduate School of Life Sciences, Tohoku University, 2–1–1 Katahira, Aoba-ku, Sendai 980–8577, Japan; 2 Department of Biochemistry and Applied Biosciences, Faculty of Agriculture, Miyazaki University, Miyazaki 889–2192, Japan; 3 National Institute for Agro-Environmental Sciences, 3–1–3, Kannondai, Tsukuba, Ibaraki 305–8604, Japan

**Keywords:** *Bradyrhizobium japonicum*, N_2_O reductase, *nosZ*, Andosol, soybean

## Abstract

*Bradyrhizobium japonicum* strains that have the *nosZ* gene, which encodes N_2_O reductase, are able to mitigate N_2_O emissions from soils (15). To examine the distribution of *nosZ* genotypes among Japanese indigenous soybean bradyrhizobia, we isolated bradyrhizobia from the root nodules of soybean plants inoculated with 32 different soils and analyzed their *nosZ* and *nodC* genotypes. The 1556 resultant isolates were classified into the *nosZ*+/*nodC*+ genotype (855 isolates) and *nosZ*−/*nodC*+ genotype (701 isolates). The 11 soil samples in which *nosZ*− isolates significantly dominated (*P* < 0.05; the χ^2^ test) were all Andosols (a volcanic ash soil prevalent in agricultural fields in Japan), whereas the 17 soil samples in which *nosZ*+ isolates significantly dominated were mainly alluvial soils (non-volcanic ash soils). This result was supported by a principal component analysis of environmental factors: the dominance of the *nosZ*− genotype was positively correlated with total N, total C, and the phosphate absorption coefficient in the soils, which are soil properties typical of Andosols. Internal transcribed spacer sequencing of representative isolates showed that the *nosZ*+ and *nosZ*− isolates of *B. japonicum* fell mainly into the USDA110 (BJ1) and USDA6 (BJ2) groups, respectively. These results demonstrated that the group lacking *nosZ* was dominant in Andosols, which can be a target soil type for an N_2_O mitigation strategy in soybean fields. We herein discussed how the *nosZ* genotypes of soybean bradyrhizobia depended on soil types in terms of N_2_O respiration selection and genomic determinants for soil adaptation.

Denitrification by the soybean endosymbiont *Bradyrhizobium japonicum* generally requires four enzymes: periplasmic nitrate reductase (Nap), nitrite reductase (Nir), nitric oxide reductase (Nor), and N_2_O reductase (Nos) (3, 11, 12, 33). These enzymes are encoded by the *napA*, *nirK*, *norCB*, and *nosZ* genes, respectively (3, 18, 20). Sameshima-Saito *et al.* (27) found two major sets of denitrification genes in indigenous soybean bradyrhizobia: a complete denitrifying group (*nosZ*+ strains) with *napA*, *nirK*, *norCB*, and *nosZ*, and an incomplete denitrifying group (*nosZ*− strains) with *napA*, *nirK*, and *norCB*. Thus, the presence of *nosZ* is variable in the genomes of soybean bradyrhizobia, although most strains possess *napA*, *nirK*, and *norCB* as core genes for denitrification from nitrate (NO_3_^−^) to nitrous oxide (N_2_O) (27). This difference was supported by comparative genomics of *B. japonicum* strains USDA110 (*nosZ*+) and USDA6 (*nosZ*−) (17).

N_2_O is a greenhouse gas and is also involved in the destruction of the ozone layer (21). N_2_O is emitted largely from agricultural lands (7, 8, 39, 40) via microbial denitrification and nitrification processes (1, 22, 30, 35). In the soybean rhizosphere, a large proportion of N_2_O is emitted during degradation of the root nodules (9, 10). On the other hand, soybean roots nodulated with the *nosZ*+ strains of *B. japonicum* are able to scavenge N_2_O, even at very low concentrations (26). This offers a promising strategy for the mitigation of N_2_O emissions from soybean fields in which native bradyrhizobia lack the *nosZ* gene (*nosZ*− strains) (8, 26). Inoculations with the *nosZ*+ strains of *B. japonicum* or *nosZ*++ (mutant strains with increased N_2_O reductase activity) (13) were found to significantly decrease N_2_O emissions from soybean rhizosphere dominated by the *nosZ*− strains of soybean bradyrhizobia (15). Although recent studies have proposed that *B. japonicum could* be subdivided into *B diazoefficiens* and *B. japonicum* (6), the conventional genus and species names of *B. japonicum* are used here (14, 15).

Genetic diversity among indigenous soybean (brady)rhizobia is influenced by soil conditions such as pH (34, 41) and by latitude (32), and analyses of the ITS (internal transcribed spacer) region between the 16S and 23S rRNA genes revealed that latitude (through its effects on temperature) is one of the main factors determining their geographical distribution in Japan (24) and the USA (31). The effects of temperature were partially verified by soil microcosm and nodulation experiments under different temperatures (23, 31). However, the relationships between soil properties, including soil type, and the diversity of indigenous soybean bradyrhizobia have not yet been elucidated in detail.

To facilitate the bradyrhizobia inoculation in soybean fields in order to mitigate N_2_O emissions (15), it is crucial to identify what determines the dominance of *nosZ*− strains in field soils. Therefore, we systematically surveyed the distributions of *nosZ*+ and *nosZ*− strains indigenous to the soils of agricultural fields in Japan, and analyzed the incidence of *nosZ* genotypes in each of the fields sampled together with soil and climate metadata. The results obtained clearly showed that *nosZ*− strains dominated in Andosol, a volcanic ash soil prevalent in Japan.

## Materials and Methods

### Soil samples

We collected 32 soil samples from agricultural fields throughout Japan (Tables 1, S2). The soil types were Andosols, Gleysols, and Fluvisols, based on the FAO soil classification (http://www.fao.org/soils-portal/soil-survey/soil-classification/en/). Fluvisols generally develop from alluvial deposits, and many Fluvisols under natural conditions are flooded periodically (16). Gleysols are wetland soils that, unless drained, are saturated with groundwater for long periods (16). Fluvisols and Gleysols in many cases generally develop from alluvial soils. We collected 32 soil samples to balance the number of Andosol and alluvial soil samples (Table 1). These soil samples were stored at 4°C until use.

### Bacterial strains and media

Two strains of *B. japonicum,* USDA110 (*nosZ*+ genotype) and T9 (*nosZ*− genotype), were used as standard strains (14). The cells were grown at 30°C in HM medium (5) supplemented with 0.1% arabinose and 0.025% (w/v) yeast extract (Difco, Detroit, MI, USA). HM medium was further supplemented with trace metals (HMM medium) for the denitrification assay (26, 28).

### Isolation of soybean bradyrhizobia

Surface-sterilized soybean seeds (*Glycine max* cv. Enrei) were germinated in sterile vermiculite for 2 d at 25°C (9, 10). Each seedling was then transplanted into a Leonard jar (one plant per jar) that contained sterile vermiculite and nitrogen-free nutrient solution (9, 10). The seedlings were then each inoculated with 1 g of soil. Plants were grown in a phytotron (Koito Industries, Tokyo, Japan) providing photosynthetically active radiation (PAR, 400–700 nm) at a photon flux density of 270 μmol m^−2^ s^−1^ for 30 d at 25/20°C with a 16-h light/8-h dark photoperiod. A nitrogen-free sterilized nutrient solution was periodically supplied to the pots (9, 10). Between 18 and 138 nodules (average 51 nodules, Table S1) were separated from the roots inoculated with each soil 30 d after the inoculation, and were surface-sterilized with 0.5% NaOCl solution. The nodules were then cut in half with sterilized razor blades, and the inner bacteroid cells were streaked on HM agar medium. After the HM agar plates had been incubated for 10 d at 30°C, single colonies were picked up from respective nodules (one colony per nodule), and transferred onto fresh HM agar plates for the following PCR analyses. Using this procedure, we collected 1639 isolates from 32 different soil samples.

### PCR analysis targeting *nosZ* and *nodC* genes

The total DNA lysate from the cultured cells was prepared as described previously (14). *nosZ* and *nodC* primers were used to detect *B. japonicum nosZ* and *nodC* (15). The primer sequences were *nosZ*-f, 5′-GACGGCGATACCATGAAAGT; *nosZ*-r, 5′-TTCTTCACTGCCTCCTCGAT; *nodC*-f, 5′-CTCCTCGCCATTTCATCACT; and *nodC*-r, 5′-CAGATATTGATCGGCGTGTG (15). The total DNA lysate was directly used as the template DNA. Ex Taq DNA polymerase (Takara, Osaka, Japan) was used for PCR amplification. The reaction mixture was incubated at 94°C for 5 min; then 30 cycles of 94°C for 30 s, 57°C for 30 s, and 72°C for 30 s; and a final 72°C for 7 min (15).

### Determination of the ITS (internal transcribed spacer) sequence

To determine the ITS sequence of representative isolates from the 32 different soil samples, we randomly selected 245 isolates from the 1556 isolates to include both *nosZ*+/− isolates based on the incidence of the *nosZ* genotype (Fig. S1, Table S3). The total DNA lysate was used as the template DNA in a 50-μL reaction mixture for PCR using ExTaq DNA polymerase. In the ITS amplification, the ITS primer set and PCR cycle conditions were the same as those described previously (23). Amplified DNA fragments were purified using the Wizard SV 96 PCR Clean-Up System and Vac-Man 96 Vacuum Manifold (Promega, Madison, WI, USA). The sequencing of amplified DNA fragments was performed by the Dragon Genomics Center at TAKARA BIO INC. (Otsu, Japan). The BigDye Terminator v3.1 Cycle Sequencing Kit (Applied Biosystems, Foster City, CA, USA) was used for the sequencing reaction, and DNA sequencing was then carried out on an ABI 3730*xl* DNA Analyzer (Applied Biosystems).

### Phylogenetic analysis

Sequences were aligned by CLUSTALW (38). On the basis of that alignment, a distance matrix was constructed using the DNADIST program from PHYLIP v. 3.66 (http://evolution.genetics.washington.edu/phylip.html) with default parameters. In order to define operational taxonomic units (OTUs) for conducting the clustering analysis, the default mothur (19) settings were used with threshold values of 99% sequence identity. In the phylogenetic analysis, sequences were aligned by CLUSTALW (38), and the neighbor-joining method was used to build a phylogenetic tree (25). The PHYLIP-format tree output was obtained by bootstrapping (http://evolution.genetics.washington.edu/phylip.html) using 1000 bootstrap trials. The trees were constructed by MEGA v. 4.0 software (37). Phylogenetic analysis used the reference strains *B. japonicum* USDA110 (Accession number AB100749), USDA122 (AB100751), USDA123 (AB100752), USDA124 (AB100753), USDA38 (AB100743), USDA6 (AB100741), T9 (AB278129), and T7 (AB278128); *B. elkanii* USDA76 (AB100747); and *Mesorhizobium loti* MAFF 303099 (BA000012).

### Physical and chemical analyses of soils

We analyzed the chemical and physical properties of soils including soil texture, pH (H_2_O), pH (KCl), total nitrogen (T-N), total carbon (T-C), C/N ratio, phosphate absorption coefficient (PAC) and available phosphate (Truog-P).

Soil samples were air-dried and passed through a 2-mm sieve. Soil particle size distribution was determined by the method of the International Society of Soil Science. Each soil sample was assigned to one of three soil coarse texture groups: 1, 2 and 3, on the basis of its position in the soil texture triangle: sandy loam (SL), loamy sand (LS), and sand (S) as group 3; sandy clay (SC), clay loam (CL), sandy clay loam (SCL), and loam (L) as group 2; and silt loam (SiL), heavy clay (HC), light clay (LiC), silty clay (SiC), and silty clay loam (SiCL) as group 1 (4).

Soil was mixed with water or 1M KCl solution at a ratio of 1 part soil (dry weight) to 2.5 parts liquid (volume); the suspension was stirred for 1 h and allowed to settle for 20 min; pH was then measured with a pH meter. T-N and T-C were measured using an NC analyzer (Sumigraph NC-220F; Sumika Chemical Analysis Service, Osaka, Japan). The C/N ratio was calculated as T-C ÷ T-N. PAC, which is used as a criterion for defining the Andosol group in the Japanese soil classification system (36), was determined by the ammonium phosphate method: In this method, 25 g of soil (dry weight) and 50 mL of 25 g L^−1^ ammonium phosphate solution (pH = 7) were placed in a 100-mL flask and shaken for 24 h. After centrifugation at 2380x*g* and filtration (Filter paper No. 5B; ADVANTEC, Tokyo, Japan), the concentration of residual phosphate in the filtrate was then determined by colorimetry. Available phosphate was analyzed by the Truog method: 0.5 g of soil (dry weight) and 400 mL 0.001 M sulfuric acid were placed in a 200-mL flask, shaken for 30 min, and then filtered. The concentration of phosphate in the filtrate was determined by colorimetry.

### Environmental metadata

We collected environmental data including temperature, precipitation, and latitudes at the soil sampling sites. Temperature was the mean annual temperature, and precipitation was the mean annual precipitation (Japan Meteorological Agency; http://www.jma.go.jp/jma/menu/menureport.html). Latitudes at the soil sampling sites were expressed in decimal format.

### Principal component analysis

PCA was performed with Canoco 4.5 software for Windows (Microcomputer Power, Ithaca, NY, USA). The ordination of soil samples was performed using soil analysis data and other environmental data as variables. Logarithms of the respective values of T-N, T-C, C/N, PAC, Truog-P, temperature, and precipitation were analyzed by PCA.

### Determination of N_2_O reductase activity

The 245 representative isolates were grown in HM broth at 30°C for 7 d. Five milliliters of each cell culture (~10^9^ cells mL^−1^) was then centrifuged at 10,000x*g* for 3 min. Collected cells were resuspended with sterilized distilled water and collected by centrifugation again. This step was repeated 3 times, and the cells were then resuspended in HMM broth at 10^9^ cells mL^−1^ in a test tube. The test tube was sealed with a butyl rubber stopper, and the headspace gas was replaced with N_2_. N_2_O gas was introduced into the headspace at a final concentration of 0.10% (v/v).

N_2_O reductase activity was determined 10 h later from the concentration of N_2_O. If the rate of N_2_O reduction was >15%, the isolates were regarded as having positive N_2_O reductase activity. N_2_O concentrations were measured by a gas chromatograph (GC-17A; Shimadzu, Kyoto, Japan) equipped with a ^63^Ni electron capture detector and CP-PoraBOND Q capillary column (internal diameter, 0.32 mm; length, 25 m; Varian, Palo Alto, CA, USA) as described previously (27, 28).

### Accession numbers of DNA sequences

The DDBJ/EMBL/GenBank accession numbers of the 16S-23S rRNA ITS regions of the isolates in the present study were from AB983890 to AB984104 and from AB985604 to AB985606 (Table S3).

## Results

### *nosZ* genotypes of indigenous soybean bradyrhizobia

**Fig. 1:**
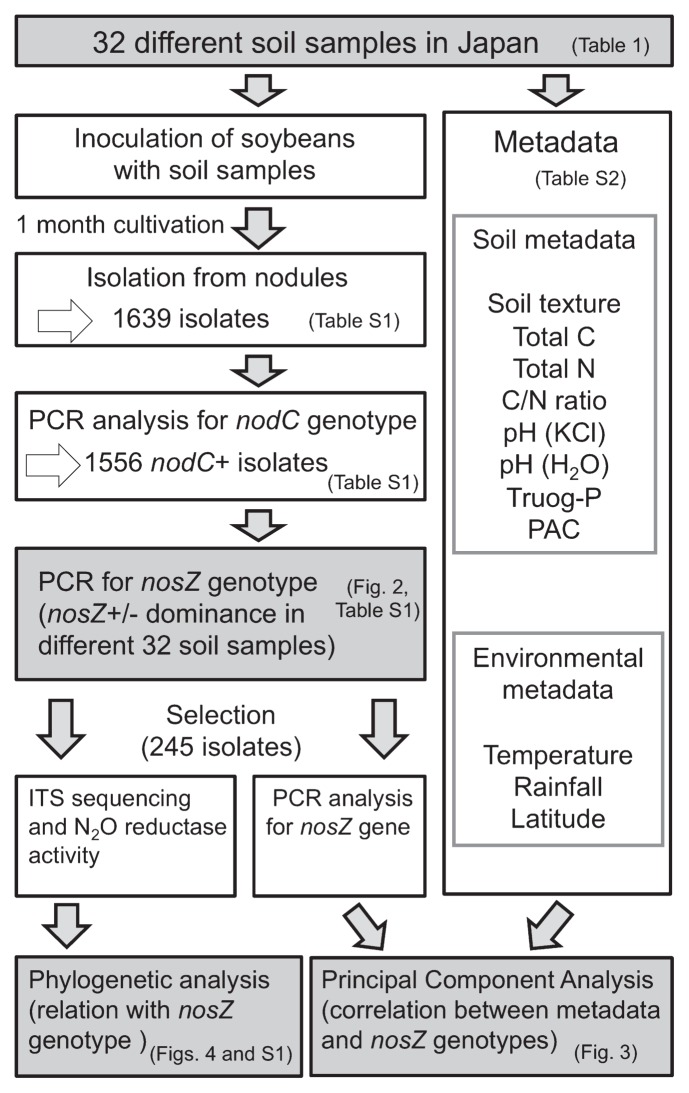
Flowchart of bradyrhizobia isolation, PCR analysis, and DNA sequence analysis.

A total of 1639 indigenous bradyrhizobia were isolated from the soybean nodules that formed following the inoculation with the 32 soil samples (Tables 1 and S1). PCR analysis specific to *nodC* of *B. japonicum* (15) suggested that 1556 isolates were *nodC*-possessing bradyrhizobia that were able to nodulate soybean plants (Fig. 1, Table S1). The subsequent diagnostic *nosZ*-specific PCR clearly showed the *nosZ* genotype because the quality of the DNA lysates had been verified by *nodC* PCR amplification (15). The isolates were classified as the *nosZ*+/*nodC*+ genotype (855 isolates) or *nosZ*−/*nodC*+ genotype (701 isolates) (Table S1).

**Fig. 2:**
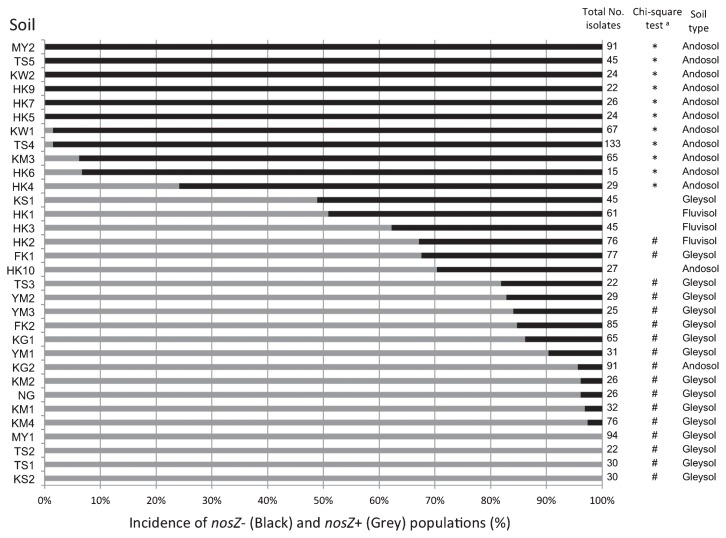
Distribution of *nosZ* genotypes in soybean bradyrhizobial populations indigenous to 32 field soils in Japan. Results are shown as the percentage of *nosZ* genotypes based on the total number of isolates. Black bars, *nosZ*− population; gray bars, *nosZ*+ population. ^a^ The genotypes of *nosZ*− (*) and *nosZ*+ (#) were significantly dominant (*P* < 0.05, by the χ^2^ test).

Results were expressed as the incidence (percentage) of *nosZ*− and *nosZ*+ isolates among the total isolates tested in each soil sample (Fig. 2). The incidence depended largely on the soil sample: six soil samples (MY2, TS5, KW2, HK5, HK7, and HK9; all Andosols) exclusively harbored *nosZ*− isolates, while four samples (MY1, TS2, TS1, and KS2; all Gleysols) harbored only *nosZ*+ isolates. The eleven soil samples in which *nosZ*− isolates significantly dominated (*P* < 0.05, by the χ^2^ test; indicated by * in Fig. 2) were all classified as Andosols. On the other hand, most of the soil samples significantly dominated by *nosZ*+ isolates (17 samples, indicated by # in Fig. 2) were classified as Gleysols (15 samples) and Fluvisols (1 sample).

Andosols, developed from volcanic ash, generally show properties distinct from other soils such as alluvial soils; *e.g.*, they are black with high water permeability, low bulk density, high carbon (humus) content, high PAC, and low pH, especially non-allophanic Andosols (36). Therefore, it is possible that the differences observed in the *nosZ* genotypes of soybean bradyrhizobia could be attributed to these soil properties because bacteria survive as soil microorganisms when host plants are absent.

### Environmental factors correlated with the *nosZ* genotype

**Fig. 3:**
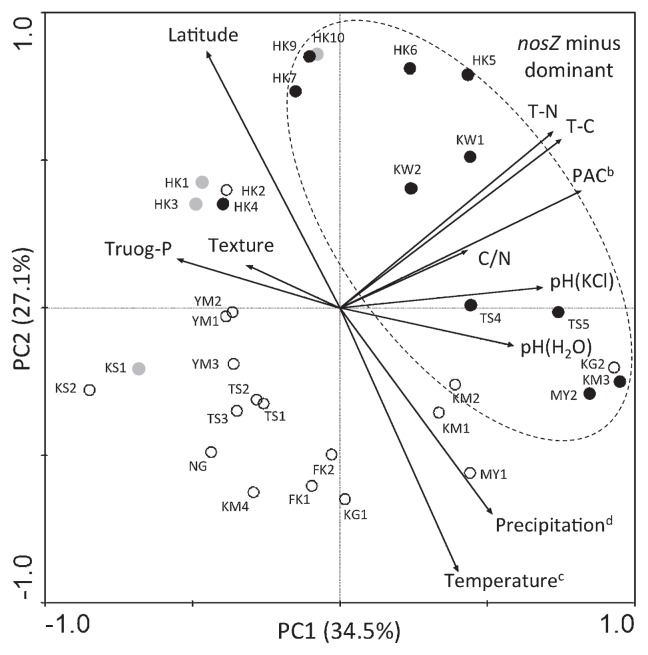
Ordination plot of the principal component analysis (PCA) of soil properties and other environmental data. The plot shows the 32 soil samples labeled with the abbreviation of the soil sampling sites (Table 1). Black-filled dots, *nosZ*− dominant soils; white dots, *nosZ*+ dominant soils; gray dots, *nosZ*+/− habitat soils (Fig. 2). The arrows show the influence of environmental variables. ^b^ PAC indicates the phosphate absorption coefficient. ^c^ Temperature is the mean annual temperature. ^d^ Precipitation is the mean annual precipitation. See Table S3 for details of these variables.

To explore the soil properties and other environmental factors that correlated with the incidence of *nosZ* genotypes, we conducted a Principal component analysis (PCA) using *nosZ* genotype data (Fig. 2) and soil & environmental metadata for each of the soil samples (Table S2). Among the 32 soil samples tested, the 11 samples dominated by *nosZ*− (Fig. 2) formed a discrete cluster (solid black dots enclosed by a dashed ellipse; Fig. 3). In the correlations between the *nosZ*− genotype with soil & environmental variables (shown by arrows in Fig. 3), the directions and lengths of the arrows indicated that T-N, T-C, and PAC were major factors that contributed to the dominance of the *nosZ*− genotype of soybean bradyrhizobia in the soil (Fig. 3). It is also likely that pH (H_2_O), pH (KCl), and C/N ratio were minor factors that contributed to its dominance.

Andosols possess high T-C and T-N contents owing to their high humus content and have a high PAC (36). The PCA results also supported Andosols favoring *nosZ*− strains.

### Phylogenetic analysis

**Fig. 4:**
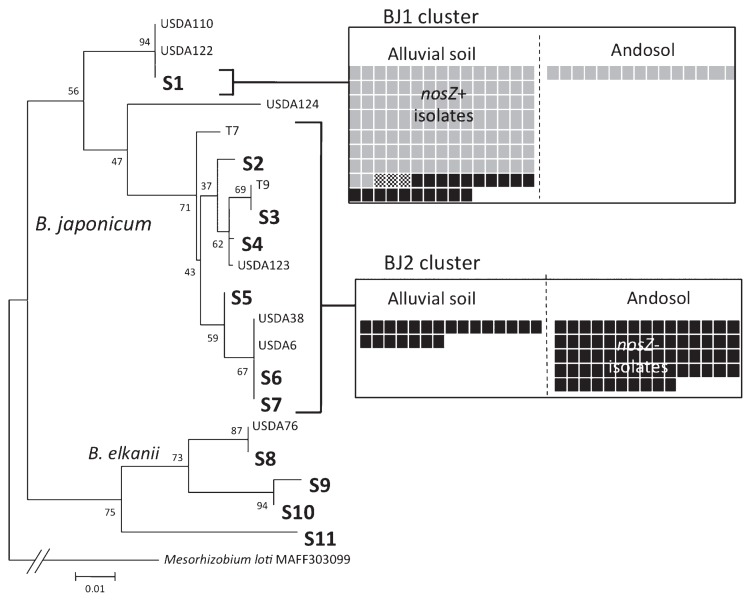
Phylogenetic distribution of *nosZ* genotypes in *B. japonicum* isolates. The phylogenetic tree was constructed from the internal transcribed spacer (ITS) region sequences. The bar indicates nucleotide base substitutions per site. S1–S11 are the operational taxonomic units (larger than 99% sequence similarity) of ITS sequences. S1 was classified as belonging to cluster BJ1 (recently proposed to be renamed *Bradyrhizobium diazoefficiens*), while OTUs from S2 to S7 were classified into cluster BJ2 (recently proposed to be renamed *Bradyrhizobium japonicum*). OTUs from S8 to S11 fell into a clade of *Bradyrhizobium elkanii*. Black squares, *nosZ*− isolates lacking N_2_O reductase activity; Gray squares, *nosZ*+ isolates with N_2_O reductase activity; Dotted squares, *nosZ*+ isolates lacking N_2_O reductase. Soil types (alluvial soils [Gleysol and Fluvisol] and Andosol) are indicated.

A phylogenetic tree was constructed from the ITS sequences of the 245 representative isolates along with several reference strains (Figs. 4 and S1, Table S3). The ITS sequences of the 245 isolates generated 11 operational taxonomic units (OTUs) with ≥99% homology. A total of 237 isolates with OTUs S1 to S7 belonged to *B. japonicum*, and 8 isolates with OTUs S8 to S11 belonged to *B. elkanii* (Figs. 4 and S1). Therefore, most isolates were *B. japonicum* (97%) possibly because our primers were designed for *B. japonicum* and had a few mismatches against the ITS regions of *B. elkanii* (15). *B. japonicum* members were subdivided into the clusters BJ1 (USDA110 group) and BJ2 (USDA6 group) (13). A recent study proposed that the members in clusters BJ1 and BJ2 could be reclassified as *B. diazoefficiens* and *B. japonicum*, respectively (6). A comparison with the reference strains USDA110 (BJ1), USDA122 (BJ1), USDA6 (BJ2), T7 (BJ2), and T9 (BJ2) indicated that the isolates with OTU S1 fell exclusively into cluster BJ1 (Fig. 4). The isolates with OTUs S2 to S7 belonged to cluster BJ2 (Fig. 4).

Cluster BJ1 comprised 145 isolates: 125 *nosZ*+ isolates (86%) and 20 *nosZ*− isolates (14%). In cluster BJ2, all 92 isolates (100%) showed the *nosZ*− genotype. Therefore, *nosZ*+ isolates were confined to BJ1 (*B. diazoefficiens*) (6). Regarding the *nosZ* genotypes, 125 *nosZ*+ and 112 *nosZ*− isolates of *B. japonicum* fell into clusters BJ1 and BJ2: 125 of the *nosZ*+ isolates (100%) fell into BJ1 and 92 of the *nosZ*− isolates (82%) fell into BJ2 (Fig. 4). These results showed that the *nosZ*+ and *nosZ*− isolates of *B. japonicum* fell mainly into the BJ1 and BJ2 groups, respectively.

By focusing on soil types, we found that alluvial soils mainly carried members of the BJ1 cluster of *B. japonicum* with *nosZ*, whereas Andosols mainly contained members of the BJ2 cluster without *nosZ*.

### N_2_O reductase activity

In the test tube assay to determine the N_2_O reductase activities (NosZ phenotype) of the 245 representative isolates (Fig. 1, Table S3), this activity was not detected in any of the 120 isolates showing the *nosZ*− genotype as determined by *nosZ*-specific PCR (black squares in Fig. S1). On the other hand, activity was detected in 122 of the 125 isolates showing the *nosZ*+ genotype (Fig. S1, Table S3). Three isolates carried *nosZ*, but did not show N_2_O reductase activity by the test tube assay, which may have been due to their very low activities or low expression of the *nos* gene cluster. However, the NosZ phenotype correlated well with the *nosZ* genotype in most isolates (242 of the 245 isolates tested; 99%).

## Discussion

Soybean bradyrhizobia with the *nosZ* gene are able to mitigate N_2_O emissions from soils (15). Our primary objective was to examine how frequently the *nosZ*− genotype appeared in soybean fields and determine whether soil properties and environmental factors affected the *nosZ* genotype of indigenous soybean bradyrhizobia. The *B. japonicum* populations lacking both *nosZ* and N_2_O reductase activity were dominant in Andosols (Figs 2 and 3). On the other hand, *nosZ*+ populations were mainly dominant in alluvial soils (Gleysols and Fluvisol) (Figs 2 and 3). Therefore, Andosol could be a target soil type for a *Bradyrhizobium*-based N_2_O mitigation strategy, at least in soybean fields in Japan (2).

We proposed two possible explanations for how the soil type (Andosol vs. alluvial soil) significantly changed the incidence of *nosZ*+ and *nosZ*− populations in indigenous *B. japonicum*: (i) selection by N_2_O respiration and (ii) genomic determinants for soil adaptation.

N_2_O respiration in *B. japonicum* USDA110 occurs under anaerobic conditions and supports anaerobic growth by using N_2_O as an electron acceptor (13, 28). In alluvial soils such as Gleysols, anaerobic environments form easily between soil particles following precipitation, and because the bulk density of Andosols is lower than that of alluvial soils, Andosols often provide a more aerobic soil environment (36). Thus, selection by N_2_O respiration would be more severe in alluvial soils than in Andosols because N_2_O respiration is induced under anaerobic conditions (28). Therefore, selection for N_2_O respiration may confer advantages to *nosZ*+ populations in terms of energy acquisition for their growth in alluvial soils. On the other hand, N_2_O respiration confers little or no advantage on Andosols, resulting in the dominance of *nosZ*− populations in Andosols.

The second explanation is the presence of unknown determinants for adaptation to different soil types. Although the sequence and gene compositions of symbiosis islands are well conserved within soybean bradyrhizobia, the core genome compositions of USDA110 (BJ1) and USDA6 (BJ2) were previously reported to differ (17). In addition, in soil microcosm experiments, a BJ1 strain (A1017ks) survived better in Fluvisol than in Andosol (19). To examine the hypothesis of “determinants for soil adaptation”, soil microcosm experiments are needed for comparisons between *nosZ*+ and *nosZ*− strains of *B. japonicum* in the future.

Phylogenetically, the *nosZ*+ and *nosZ*− isolates of *B. japonicum* fell mainly into BJ1 and BJ2 clusters, respectively (Fig. 4). These results were consistent with previous findings (14, 27). Genomic comparisons between USDA110 (BJ1) and USDA6 (BJ2) indicated that the position of the *nos* gene cluster (*nosRZDFYLX*) on the USDA110 genome corresponded to a typical genomic island flanking to trnS-GGA on the USDA6 genome, suggesting that the *nos* gene cluster is a mobile genomic island on the USDA110 genome (17). In the present study, the *nosZ*+ genotype was confined to the BJ1 cluster (Fig. 4). Thus, the presumptive “*nos* genomic island” was likely to have been acquired during the evolution of the BJ1 lineage.

In the present study, we systemically obtained and characterized many isolates of soybean bradyrhizobia. Sanchez *et al.* (28) recently found that the nitrate-sensing NasST system controlled *nosZ* gene expression in *B. japonicum*: Artificial *nasS* mutations both induced *nosZ* expression and enhanced the N_2_O reductase activity of *B. japonicum* via NasT (28). Our culture collection of soybean bradyrhizobia may be used for screening of *nasS* natural mutations. In addition to contributing to research on denitrification genes and their regulatory genes, this culture collection will contribute to research on the interactions between soybean plants and bradyrhizobia as a bioresource.

## Supplementary materials



## Figures and Tables

**Table 1:** Soils used in the present study.

Soil	Soil type	Prefecture	Latitude (°N)	Longitude (°E)	Soil sampling date	Cultivation history^a^	Source
HK1	Fluvisol	Hokkaido	43.07	141.35	Feb-08	Rotation, +NPK	T. Ezawa
HK2	Fluvisol	Hokkaido	43.07	141.35	Feb-08	Rotation, −N	T. Ezawa
HK3	Fluvisol	Hokkaido	43.07	141.35	Feb-08	Rotation, −P	T. Ezawa
HK4	Andosol	Hokkaido	42.91	143.05	Sep-07	Yam	T. Kaji
HK5	Andosol	Hokkaido	43.08	142.84	Aug-07	Carrot	T. Kaji
HK6	Andosol	Hokkaido	43.08	142.84	Aug-07	Carrot	T. Kaji
HK7	Andosol	Hokkaido	42.99	143.20	Aug-07	Onion	T. Kaji
HK9	Andosol	Hokkaido	42.99	143.20	Sep-07	Asparagus	T. Kaji
HK10	Andosol	Hokkaido	42.99	143.20	Sep-07	Asparagus	T. Kaji
KW1	Andosol	Miyagi	38.74	140.76	Jul-09	Soybean	M. Saito
KW2	Andosol	Miyagi	38.74	140.76	Jul-09	No cultivation	M. Saito
KS1	Gleysol	Miyagi	38.46	141.09	Oct-07	Soybean	M. Itakura
KS2	Gleysol	Miyagi	38.46	141.09	Oct-07	Paddy field	M. Itakura
YM1	Gleysol	Yamagata	38.24	140.37	Aug-07	Soybean	H. Shiono
YM2	Gleysol	Yamagata	38.24	140.37	Aug-07	Soybean	H. Shiono
YM3	Gleysol	Yamagata	38.24	140.37	Aug-07	Soybean	H. Shiono
NG	Gleysol	Niigata	37.44	138.87	Sep-07	Soybean	Y. Shiratori
TS1	Gleysol	Ibaraki	36.03	140.11	Aug-07	Soybean	M. Hayatsu
TS2	Gleysol	Ibaraki	36.03	140.11	Aug-07	Upland rice	M. Hayatsu
TS3	Gleysol	Ibaraki	36.03	140.11	Aug-07	Paddy rice	M. Hayatsu
TS4	Andosol	Ibaraki	36.03	140.11	Oct-08	Soybean	M. Hayatsu
TS5	Andosol	Ibaraki	36.03	140.11	Oct-08	Soybean	M. Hayatsu
FK1	Gleysol	Fukuoka	33.61	130.46	Jun-09	Soybean	T. Yamakawa
FK2	Gleysol	Fukuoka	33.21	130.43	Jun-09	Soybean, Wheat	M. Araki
KM1	Gleysol	Kumamoto	32.89	130.77	Jun-09	Soybean	H. Mizukami
KM2	Gleysol	Kumamoto	32.89	130.77	Jun-09	Soybean	H. Mizukami
KM3	Andosol	Kumamoto	32.89	130.77	Jun-09	Soybean	M. Matsumori
KM4	Gleysol	Kumamoto	32.76	130.76	Jun-09	Paddy rice	N. Gunjikake
MY1	Gleysol	Miyazaki	32.00	131.47	Jun-09	Soybean	Y. Saeki
MY2	Andosol	Miyazaki	31.83	131.41	Jun-09	Soybean	Y. Saeki
KG1	Gleysol	Kagoshima	31.39	130.38	Jun-09	Paddy rice	T. Mochida
KG2	Andosol	Kagoshima	31.44	130.92	Jun-09	Soybean	T. Mochida

a Rotation: Crop rotation of soybean, corn, oats, beets, and sunflower. +NPK: N, P and K fertilizers were fully applied to the upland field as macronutrients. −N: without N fertilizer. −P: without P fertilizer.
