# Supplementary material for: Relationship Between Soil Type and N2O Reductase Genotype (nosZ) of Indigenous Soybean Bradyrhizobia: nosZ-minus Populations are Dominant in Andosols
Source: Microbes Environ. 2014 Dec 3;29(4):420–6. doi: 10.1264/jsme2.ME14130 (PMC4262367; doi:10.1264/jsme2.ME14130)
Supplement: Supplementary file 1 [file 29_420_s1.pdf]

Supplementary materials

Correlation between soil type and N_2O reductase genotype (*nosZ*) of indigenous soybean bradyrhizobia: *nosZ*-minus populations are dominant in Andosols

Yoko Shiina,[1] Manabu Itakura,[1] Hyunseok Choi,[1] Yuichi Saeki,[2] Masahito Hayatsu[3] and Kiwamu Minamisawa[1]*

[1] Graduate School of Life Sciences, Tohoku University, 2-1-1 Katahira, Aoba-ku, Sendai 980-8577, Japan; [2] Department of Biochemistry and Applied Biosciences, Faculty of Agriculture, Miyazaki University, Miyazaki 889-2192, Japan; [3] National Institute for Agro-Environmental Sciences, 3-1-3, Kannondai, Tsukuba, Ibaraki 305-8604, Japan

Table S1. PCR amplification for *nodC* and *nosZ* genes of soybean bradyrhizobial isolates[a]

Soil name	Number of total isolates	*nodC* PCR				*nosZ* PCR			
		Positive		Negative		Positive		Negative	
		Number	Percentage	Number	Percentage	Number	Percentage	Number	Percentage
HK1	76	61	80%	15	20%	31	51%	30	49%
HK2	77	76	99%	1	1%	51	67%	25	33%
HK3	78	45	58%	33	42%	28	62%	17	38%
HK4	29	29	100%	0	0%	7	24%	22	76%
HK5	24	24	100%	0	0%	0	0%	24	100%
HK6	18	15	83%	3	17%	1	7%	14	93%
HK7	27	26	96%	1	4%	0	0%	26	100%
HK9	27	22	81%	5	19%	0	0%	22	100%
HK10	29	27	93%	2	7%	19	70%	8	30%
KW1	69	67	97%	2	3%	1	1%	66	99%
KW2	25	24	96%	1	4%	0	0%	24	100%
KS1	46	45	98%	1	2%	22	49%	23	51%
KS2	30	30	100%	0	0%	30	100%	0	0%
YM1	32	31	97%	1	3%	28	90%	3	10%
YM2	29	29	100%	0	0%	24	83%	5	17%
YM3	25	25	100%	0	0%	21	84%	4	16%
NG	26	26	100%	0	0%	25	96%	1	4%
TS1	30	30	100%	0	0%	30	100%	0	0%
TS2	22	22	100%	0	0%	22	100%	0	0%
TS3	22	22	100%	0	0%	18	82%	4	18%
TS4	138	133	96%	5	4%	2	2%	131	98%
TS5	46	45	98%	1	2%	0	0%	45	100%
FK1	86	77	90%	9	10%	52	68%	25	32%
FK2	87	85	98%	2	2%	72	85%	13	15%
KM1	33	32	97%	1	3%	31	97%	1	3%
KM2	26	26	100%	0	0%	25	96%	1	4%
KM3	65	65	100%	0	0%	4	6%	61	94%
KM4	76	76	100%	0	0%	74	97%	2	3%
MY1	94	94	100%	0	0%	94	100%	0	0%
MY2	91	91	100%	0	0%	0	0%	91	100%
KG1	65	65	100%	0	0%	56	86%	9	14%
KG2	91	91	100%	0	0%	87	96%	4	4%
Total	1639	1556	95%	83	5%	855	55%	701	45%

[a] *nodC* positive isolates were tested for *nosZ* PCR (Fig. 1)

Table S2. Environmental data for Principal Component Analysis (PCA).

Soil	Soil type	Soil texture				pH	pH	T-N	T-C	P abs. coeff	Truog-P	Temperature	Precipitation	Latitude	Longitude
		Sand (%)	Silt (%)	Clay (%)	Group[a]	(H_2O)	(KCl)	(%)	(%)	(mg/100g)	(mg/100g)	(°C)	(ml)		
HK1	Fluvisol	40.7	36.9	22.4	2	5.3	4.5	0.22	3.6	613	42.2	8.5	1128	43.07	141.35
HK2	Fluvisol	39.4	36.6	24.0	2	5.6	4.7	0.20	3.2	732	47.2	8.5	1128	43.07	141.35
HK3	Fluvisol	42.4	36.5	21.1	2	5.1	4.2	0.21	3.2	629	4.0	8.5	1128	43.07	141.35
HK4	Andosol	56.2	42.0	1.8	2	5.8	4.8	0.16	1.7	1303	3.8	6.0	969	42.91	143.05
HK5	Andosol	53.0	41.1	5.8	2	6.3	5.9	0.54	7.3	1801	8.5	6.5	1139	43.08	142.84
HK6	Andosol	49.6	41.1	9.4	2	5.8	5.2	0.58	7.8	1789	6.4	6.5	1139	43.08	142.84
HK7	Andosol	45.6	50.0	4.4	2	5.7	4.9	0.45	5.3	1181	24.9	6.5	920	42.99	143.20
HK9	Andosol	45.0	48.9	6.1	2	5.5	5.0	0.51	6.8	1313	64.1	6.5	920	42.99	143.20
HK10	Andosol	50.9	43.4	5.7	2	5.2	4.7	0.55	7.0	1656	9.2	6.5	920	42.99	143.20
KW1	Andosol	37.7	51.1	11.2	2	6.3	5.1	0.56	9.8	1544	39.9	10.4	1417	38.74	140.76
KW2	Andosol	52.2	44.8	3.0	2	5.3	4.5	0.42	7.8	1461	1.0	10.4	1417	38.74	140.76
KS1	Gleysol	64.4	18.0	17.6	2	5.4	4.3	0.08	1.2	568	10.8	11.0	1153	38.46	141.09
KS2	Gleysol	75.3	12.7	12.2	3	5.7	4.7	0.11	0.9	403	10.3	11.0	1153	38.46	141.09
YM1	Gleysol	42.2	54.9	2.8	2	5.3	4.6	0.17	2.2	650	3.4	11.5	1125	38.24	140.37
YM2	Gleysol	50.2	47.2	2.6	2	5.4	4.7	0.19	2.4	566	4.6	11.5	1125	38.24	140.37
YM3	Gleysol	54.1	41.8	4.1	2	6.3	5.3	0.11	1.7	307	25.6	11.5	1125	38.24	140.37
NG	Gleysol	47.3	30.7	22.0	2	5.6	4.6	0.12	1.2	705	12.9	12.7	2309	37.44	138.87
TS1	Gleysol	34.0	30.0	36.0	1	5.6	4.7	0.16	1.9	542	5.4	13.5	1236	36.03	140.11
TS2	Gleysol	34.0	30.0	36.0	1	5.7	4.7	0.16	1.9	549	13.3	13.5	1236	36.03	140.11
TS3	Gleysol	34.0	30.0	36.0	1	5.5	4.7	0.16	1.9	394	7.1	13.5	1236	36.03	140.11
TS4	Andosol	18.9	26.3	54.8	1	5.8	5.0	0.37	4.2	1978	0.3	13.5	1236	36.03	140.11
TS5	Andosol	18.9	26.3	54.8	1	6.7	5.9	0.37	4.2	1799	0.6	13.5	1236	36.03	140.11
FK1	Fluvisol	33.1	25.3	41.7	2	6.0	5.0	0.13	2.0	464	10.8	16.8	1753	33.61	130.46
FK2	Gleysol	17.0	80.4	2.6	2	6.2	5.3	0.23	2.2	638	50.8	16.6	1780	33.21	130.43
KM1	Gleysol	42.9	55.2	1.9	2	6.1	5.5	0.23	3.1	1190	8.7	15.4	1989	32.89	130.77
KM2	Gleysol	42.9	41.1	16.0	2	6.1	5.4	0.33	4.2	1184	10.9	15.4	1989	32.89	130.77
KM3	Andosol	46.7	51.6	1.7	1	5.8	5.3	0.44	7.3	2181	0.3	17.2	2353	32.89	130.77
KM4	Gleysol	30.0	68.5	1.5	2	5.0	4.0	0.20	2.4	672	9.7	17.5	2495	32.76	130.76
MY1	Gleysol	53.3	29.3	17.5	2	6.1	5.4	0.20	3.0	1360	9.8	17.4	2797	32.00	131.47
MY2	Andosol	48.6	35.4	16.0	2	6.0	5.1	0.50	7.3	2218	0.7	17.4	2797	31.83	131.41
KG1	Gleysol	63.8	34.3	2.0	2	5.6	4.6	0.20	2.5	851	7.1	17.7	2385	31.39	130.38
KG2	Andosol	59.1	37.5	3.33	2	6.1	5.2	0.43	9.6	1875	1.6	17.3	2581	31.44	130.92

a Each soil sample was assigned to one of three soil coarse texture groups 1, 2 and 3 on the basis of its position in the soil texture triangle: sandy loam (SL), loamy sand (LS), and sand (S) as group 3; sandy clay (SC), clay loam (CL), sandy clay loam (SCL), and loam (L) as group 2; and silt loam (SiL), heavy clay (HC), light clay (LiC), silty clay (SiC), and silty clay loam (SiCL) as group 1.

Table S3. List of isolates using phylogenetic analysis

Soil name	Number of isolates	Isolates	*nosZ* genotype	NosZ phenotype	Accession number		OTU name in Fig. 4
					16S-23S ITS	16S rDNA	
HK1	9	HK1-1	-	-	AB983863	AB984105	S3
		HK1-2	+	+	AB983864	AB984106	S1
		HK1-3	-	-	AB983865	AB984107	S3
		HK1-7	+	+	AB983866	AB984108	S1
		HK1-9	+	+	AB983867	AB984109	S1
		HK1-31	+	+	AB983868	AB984110	S1
		HK1-32	+	+	AB983869	-	S1
		HK1-37	-	-	AB983870	AB984111	S4
		HK1-39	-	-	AB983871	AB984112	S1
HK2	7	HK2-2	+	+	AB983872	AB984113	S1
		HK2-3	+	+	AB983873	AB984114	S1
		HK2-5	+	+	AB983874	AB984115	S1
		HK2-6	+	+	AB983875	AB984116	S1
		HK2-8	+	+	AB983876	AB984117	S1
		HK2-9	+	+	AB983877	AB984118	S1
		HK2-27	+	+	AB983878	AB984119	S1
HK3	10	HK3-4	-	-	AB983879	AB984120	S10
		HK3-6	-	-	AB983880	AB984121	S10
		HK3-7	+	+	AB983881	AB984122	S1
		HK3-8	+	+	AB983882	AB984123	S1
		HK3-11	+	+	AB983883	AB984124	S1
		HK3-13	+	+	AB983884	AB984125	S1
		HK3-21	+	+	AB983885	AB984126	S1
		HK3-25	+	+	AB983886	AB984127	S1
		HK3-39	-	-	AB983887	AB984128	S1
		HK3-40	-	-	AB983888	AB984129	S1
HK4	12	HK4-1	-	-	AB983889	AB984130	S2
		HK4-5	-	-	AB985604	AB985607	S3
		HK4-6	-	-	AB983890	AB984131	S3
		HK4-8	-	-	AB983891	AB984132	S3
		HK4-10	-	-	AB983892	AB984133	S11
		HK4-11	-	-	AB983893	AB984134	S3
		HK4-12	-	-	AB985605	AB985608	S3
		HK4-14	-	-	AB983894	AB984135	S2
		HK4-15	-	-	AB983895	AB984136	S2
		HK4-16	-	-	AB983896	AB984137	S3
		HK4-19	-	-	AB983897	AB984138	S2
		HK4-20	-	-	AB983898	AB984139	S3
HK5	7	HK5-1	-	-	AB983899	AB984140	S11
		HK5-3	-	-	AB983900	AB984141	S4
		HK5-4	-	-	AB983901	AB984142	S2
		HK5-7	-	-	AB983902	AB984143	S2
		HK5-8	-	-	AB983903	AB984144	S3
		HK5-25	-	-	AB983904	AB984145	S3
		HK5-26	-	-	AB983905	AB984146	S3
HK6	7	HK6-1	-	-	AB983906	AB984147	S3
		HK6-2	-	-	AB983907	AB984148	S4
		HK6-5	-	-	AB983908	AB984149	S3
		HK6-7	-	-	AB983909	AB984150	S3
		HK6-8	-	-	AB983910	AB984151	S3
		HK6-9	-	-	AB983911	AB984152	S3
		HK6-11	+	+	AB983912	AB984153	S2
HK7	2	HK7-6	-	-	AB983913	AB984154	S2
		HK7-8	-	-	AB983914	AB984155	S2
HK9	5	HK9-2	-	-	AB983915	AB984156	S2
		HK9-3	-	-	AB983916	AB984157	S2
		HK9-4	-	-	AB983917	AB984158	S3
		HK9-11	-	-	AB983918	AB984159	S3
		HK9-12	-	-	AB983919	AB984160	S4
HK10	11	HK10-2	+	+	AB983920	AB984161	S1
		HK10-3	+	+	AB983921	AB984162	S1
		HK10-5	-	-	AB983922	AB984163	S3
		HK10-7	+	+	AB983923	AB984164	S1
		HK10-8	-	-	AB983924	AB984165	S3
		HK10-9	+	+	AB983925	AB984166	S1

		HK10-10	-	-	AB983926	AB984167	S4
		HK10-11	+	+	AB985606	AB985609	S1
		HK10-12	-	-	AB983927	AB984168	S3
		HK10-13	+	+	AB983928	AB984169	S1
		HK10-14	+	+	AB983929	AB984170	S1
KW1	9	KW1-1	-	-	AB983930	AB984171	S3
		KW1-2	-	-	AB983931	AB984172	S3
		KW1-3	-	-	AB983932	AB984173	S3
		KW1-4	-	-	AB983933	AB984174	S3
		KW1-6	-	-	AB983934	AB984175	S6
		KW1-8	-	-	AB983935	AB984176	S6
		KW1-9	-	-	AB983936	AB984177	S6
		KW1-52	-	-	AB983937	AB984178	S3
		KW1-63	-	-	AB983938	AB984179	S2
KW2	6	KW2-1	-	-	AB983939	AB984180	S3
		KW2-2	-	-	AB983940	AB984181	S3
		KW2-3	-	-	AB983941	AB984182	S3
		KW2-4	-	-	AB983942	AB984183	S3
		KW2-5	-	-	AB983943	AB984184	S3
		KW2-6	-	-	AB983944	AB984185	S3
KS1	10	KS1-2	-	-	AB983945	AB984186	S2
		KS1-4	-	-	AB983946	AB984187	S1
		KS1-5	-	-	AB983947	AB984188	S1
		KS1-7	+	+	AB983948	AB984189	S1
		KS1-9	-	-	AB983949	AB984190	S1
		KS1-12	-	-	AB983950	AB984191	S4
		KS1-13	-	-	AB983951	AB984192	S4
		KS1-16	+	+	AB983952	AB984193	S1
		KS1-23	-	-	AB983953	AB984194	S1
		KS1-25	-	-	AB983954	AB984195	S1
KS2	7	KS2-1	+	+	AB983955	AB984196	S1
		KS2-2	+	+	AB983956	AB984197	S1
		KS2-3	+	+	AB983957	AB984198	S1
		KS2-4	+	+	AB983958	AB984199	S1
		KS2-5	+	+	AB983959	AB984200	S1
		KS2-6	+	+	AB983960	AB984201	S1
		KS2-21	+	+	AB983961	AB984202	S1
YM1	9	YM1-1	+	+	AB983962	AB984203	S1
		YM1-2	-	-	AB983963	AB984204	S1
		YM1-3	+	+	AB983964	AB984205	S1
		YM1-4	+	+	AB983965	AB984206	S1
		YM1-5	+	+	AB983966	AB984207	S1
		YM1-6	+	+	AB983967	AB984208	S1
		YM1-7	+	+	AB983968	AB984209	S1
		YM1-23	-	-	AB983969	AB984210	S1
		YM1-24	-	-	AB983970	AB984211	S1
YM2	9	YM2-1	+	+	AB983971	AB984212	S1
		YM2-2	+	+	AB983972	AB984213	S1
		YM2-3	+	+	AB983973	AB984214	S1
		YM2-5	+	+	AB983974	AB984215	S1
		YM2-6	+	+	AB983975	AB984216	S1
		YM2-7	+	+	AB983976	AB984217	S1
		YM2-8	-	-	AB983977	AB984218	S1
		YM2-19	-	-	AB983978	AB984219	S1
		YM2-30	-	-	AB983979	AB984220	S1
YM3	9	YM3-2	+	+	AB983980	AB984221	S1
		YM3-4	+	+	AB983981	AB984222	S1
		YM3-6	+	+	AB983982	AB984223	S1
		YM3-7	+	+	AB983983	AB984224	S1
		YM3-8	+	+	AB983984	AB984225	S1
		YM3-10	-	-	AB983985	AB984226	S7
		YM3-11	+	+	AB983986	AB984227	S1
		YM3-14	-	-	AB983987	AB984228	S7
		YM3-24	-	-	AB983988	AB984229	S8
NG	6	NG-1	+	+	AB983989	AB984230	S1
		NG-2	+	+	AB983990	AB984231	S1
		NG-3	-	-	AB983991	AB984232	S1
		NG-4	+	+	AB983992	AB984233	S1
		NG-5	+	+	AB983993	AB984234	S1
		NG-7	+	+	AB983994	AB984235	S1
TS1	6	TS1-1	+	+	AB983995	AB984236	S1

		TS1-2	+	+	AB983996	AB984237	S1
		TS1-3	+	+	AB983997	AB984238	S1
		TS1-4	+	+	AB983998	AB984239	S1
		TS1-5	+	+	AB983999	AB984240	S1
		TS1-6	+	+	AB984000	AB984241	S1
TS2	5	TS2-1	+	+	AB984001	AB984242	S1
		TS2-3	+	+	AB984002	AB984243	S1
		TS2-7	+	+	AB984003	AB984244	S1
		TS2-9	+	+	AB984004	AB984245	S1
		TS2-10	+	+	AB984005	AB984246	S1
TS3	10	TS3-3	+	+	AB984006	AB984247	S1
		TS3-4	+	+	AB984007	AB984248	S1
		TS3-5	+	+	AB984008	AB984249	S1
		TS3-6	+	+	AB984009	AB984250	S1
		TS3-7	+	+	AB984010	AB984251	S1
		TS3-9	+	+	AB984011	AB984252	S1
		TS3-12	+	+	AB984012	AB984253	S1
		TS3-15	+	+	AB984013	AB984254	S1
		TS3-16	-	-	AB984014	AB984255	S7
		TS3-19	+	+	AB984015	AB984256	S1
TS4	5	TS4-11	-	-	AB984016	AB984257	S7
		TS4-12	-	-	AB984017	AB984258	S7
		TS4-21	-	-	AB984018	AB984259	S5
		TS4-125	+	+	AB984019	AB984260	S1
		TS4-229	+	+	AB984020	AB984261	S1
TS5	2	TS5-2	-	-	AB984021	AB984262	S7
		TS5-6	-	-	AB984022	AB984263	S7
FK1	10	FK1-2	-	-	AB984023	AB984264	S7
		FK1-3	-	-	AB984024	AB984265	S7
		FK1-4	-	-	AB984025	AB984266	S7
		FK1-5	-	-	AB984026	AB984267	S7
		FK1-6	+	-	AB984027	AB984268	S1
		FK1-7	+	-	AB984028	AB984269	S1
		FK1-8	+	+	AB984029	AB984270	S1
		FK1-9	-	-	AB984030	AB984271	S7
		FK1-10	-	-	AB984031	AB984272	S7
		FK1-11	+	+	AB984032	AB984273	S1
FK2	12	FK2-1	+	+	AB984033	AB984274	S1
		FK2-2	+	+	AB984034	AB984275	S1
		FK2-3	+	+	AB984035	AB984276	S1
		FK2-4	+	+	AB984036	AB984277	S1
		FK2-5	+	+	AB984037	AB984278	S1
		FK2-6	+	+	AB984038	AB984279	S1
		FK2-27	-	-	AB984039	AB984280	S7
		FK2-28	-	-	AB984040	AB984281	S1
		FK2-36	-	-	AB984041	AB984282	S7
		FK2-38	-	-	AB984042	AB984283	S8
		FK2-44	-	-	AB984043	AB984284	S7
		FK2-46	-	-	AB984044	AB984285	S7
KM1	7	KM1-1	+	+	AB984045	AB984286	S1
		KM1-2	+	+	AB984046	AB984287	S1
		KM1-3	+	+	AB984047	AB984288	S1
		KM1-4	+	-	AB984048	AB984289	S1
		KM1-5	+	+	AB984049	AB984290	S1
		KM1-6	+	+	AB984050	AB984291	S1
		KM1-13	-	-	AB984051	AB984292	S7
KM2	7	KM2-1	+	+	AB984052	AB984293	S1
		KM2-3	+	+	AB984053	AB984294	S1
		KM2-5	+	+	AB984054	AB984295	S1
		KM2-6	+	+	AB984055	AB984296	S1
		KM2-7	+	+	AB984056	AB984297	S1
		KM2-8	+	+	AB984057	AB984298	S1
		KM2-20	-	-	AB984058	AB984299	S7
KM3	10	KM3-1	-	-	AB984059	AB984300	S7
		KM3-2	-	-	AB984060	AB984301	S7
		KM3-4	+	+	AB984061	AB984302	S1
		KM3-5	-	-	AB984062	AB984303	S7
		KM3-6	-	-	AB984063	AB984304	S7
		KM3-7	-	-	AB984064	AB984305	S7
		KM3-8	-	-	AB984065	AB984306	S7
		KM3-9	-	-	AB984066	AB984307	S7

		KM3-11	+	+	AB984067	AB984308	S1
		KM3-58	+	+	AB984068	AB984309	S1
KM4	8	KM4-1	+	+	AB984069	AB984310	S1
		KM4-2	+	+	AB984070	AB984311	S1
		KM4-3	+	+	AB984071	AB984312	S1
		KM4-4	+	+	AB984072	AB984313	S1
		KM4-5	+	+	AB984073	AB984314	S1
		KM4-6	+	+	AB984074	AB984315	S1
		KM4-52	-	-	AB984075	AB984316	S1
		KM4-65	-	-	AB984076	AB984317	S1
MY1	6	MY1-1	+	+	AB984077	AB984318	S1
		MY1-2	+	+	AB984078	AB984319	S1
		MY1-3	+	+	AB984079	AB984320	S1
		MY1-4	+	+	AB984080	AB984321	S1
		MY1-5	+	+	AB984081	AB984322	S1
		MY1-6	+	+	AB984082	AB984323	S1
MY2	6	MY2-1	-	-	AB984083	AB984324	S6
		MY2-2	-	-	AB984084	AB984325	S5
		MY2-3	-	-	AB984085	AB984326	S7
		MY2-4	-	-	AB984086	AB984327	S5
		MY2-5	-	-	AB984087	AB984328	S5
		MY2-6	-	-	AB984088	AB984329	S6
KG1	11	KG1-1	+	+	AB984089	AB984330	S8
		KG1-2	+	+	AB984090	AB984331	S1
		KG1-3	+	+	AB984091	AB984332	S1
		KG1-5	+	+	AB984092	AB984333	S1
		KG1-6	-	-	AB984093	AB984334	S7
		KG1-7	-	-	AB984094	AB984335	S1
		KG1-8	-	-	AB984095	AB984336	S1
		KG1-10	+	+	AB984096	AB984337	S1
		KG1-11	+	+	AB984097	AB984338	S1
		KG1-13	+	+	AB984098	AB984339	S1
		KG1-50	-	-	AB984099	AB984340	S9
KG2	5	KG2-5	+	+	AB984100	AB984341	S1
		KG2-6	+	+	AB984101	AB984342	S1
		KG2-55	-	-	AB984102	AB984343	S1
		KG2-80	-	-	AB984103	AB984344	S7
		KG2-88	-	-	AB984104	AB984345	S7
Total	245						11 OTUs

Light gray column indicates *nosZ*+ isolates lacking N_2O reductase activity.

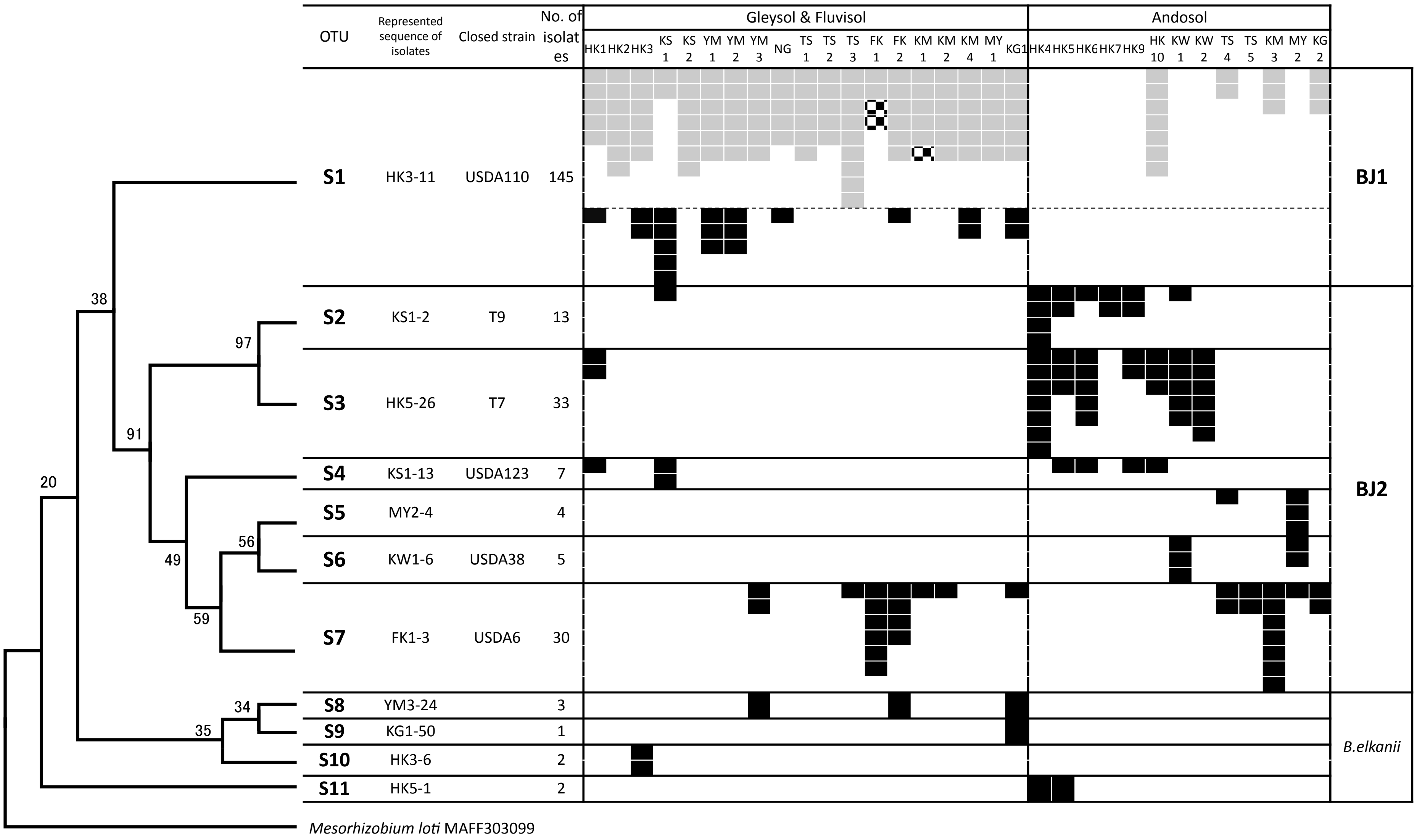

Fig. S1. Detailed phylogenic distribution of *nosZ* genotype in soybean bradyrhizobial isolates from 32 soils. Phylogenetic tree was constructed on the base of ITS region sequences (Fig. 4). S1~S11 show operational taxonomic unit (99%) of ITS sequences. OTU S1 belonged to BJ1 cluster (recently renamed *Bradyrhizobium diazoefficiens*), while OTUs from S2 to S7 were classified into BJ2 cluster (recently renamed *Bradyrhizobium japonicum*). OTUs from S8 to S11 fell into a clade of *Bradyrhizobium elkanii*. Black squares indicate *nosZ*- isolates lacking N_2O reductase activity, gray squares indicate *nosZ*+ isolates with N_2O reductase activity, dotted squares indicate *nosZ*+ isolates lacking N_2O reductase activity.
Soil types (alluvial soil (Glysol and Fluvisol) and Andosol) are indicated.
